# Computational causal discovery for post-traumatic stress in police officers

**DOI:** 10.1038/s41398-020-00910-6

**Published:** 2020-08-11

**Authors:** Glenn N. Saxe, Sisi Ma, Leah J. Morales, Isaac R. Galatzer-Levy, Constantin Aliferis, Charles R. Marmar

**Affiliations:** 1 Department of Child and Adolescent Psychiatry, New York University School of Medicine, New York, NY USA; 2 Institute of Health Informatics, University of Minnesota School of Medicine, Minneapolis, MN USA; 3 Perlmutter Cancer Center, New York University School of Medicine, New York, NY USA; 4 Department of Psychiatry, New York University School of Medicine, New York, NY USA

**Keywords:** Clinical genetics, Neuroscience, Human behaviour, Psychiatric disorders

## Abstract

This article reports on a study aimed to elucidate the complex etiology of post-traumatic stress (PTS) in a longitudinal cohort of police officers, by applying rigorous computational causal discovery (CCD) methods with observational data. An existing observational data set was used, which comprised a sample of 207 police officers who were recruited upon entry to police academy training. Participants were evaluated on a comprehensive set of clinical, self-report, genetic, neuroendocrine and physiological measures at baseline during academy training and then were re-evaluated at 12 months after training was completed. A data-processing pipeline—the *Protocol for Computational Causal Discovery in Psychiatry (PCCDP)*—was applied to this data set to determine a causal model for PTS severity. A causal model of 146 variables and 345 bivariate relations was discovered. This model revealed 5 direct causes and 83 causal pathways (of four steps or less) to PTS at 12 months of police service. Direct causes included single-nucleotide polymorphisms (SNPs) for the *Histidine Decarboxylase (HDC)* and *Mineralocorticoid Receptor (MR)* genes, acoustic startle in the context of low perceived threat during training, peritraumatic distress to incident exposure during first year of service, and general symptom severity during training at 1 year of service. The application of CCD methods can determine variables and pathways related to the complex etiology of PTS in a cohort of police officers. This knowledge may inform new approaches to treatment and prevention of critical incident related PTS.

## Introduction

This article reports on a study that applies computational causal discovery (CCD) methods to a unique longitudinal observational data set, to determine causal factors for post-traumatic stress (PTS) related to police duty critical incident exposure. The identification of causal factors controlling the expression of PTS is necessary to advance intervention for PTS. Advances in intervention requires research that identifies causal factors^1,2^, but the scientific literature that would inform the identification of causes are almost exclusively based on the application of correlational methods to observational data. Causal inferences from such research will frequently be in error^1,2^. Experimental etiologic research can infer causes, but such research—for all practical purposes—cannot be conducted with humans (e.g., randomizing a subject to a trauma condition). Animal experimental studies have provided important causal knowledge about such aspects as fear conditioning and attachment systems likely involved in PTS^3,4^. There are, however, obvious problems with exclusively relying on animal studies to derive knowledge on causal factors^5,6^. Thus, advances in intervention will need to come from establishment of alternative methods to experimental research to determine causal factors. The present study was conducted to explore application of CCD methods for this purpose. There is an emerging literature identifying the importance of this problem of causal inference for research in psychiatry^7–10^.

There is an extensive literature validating the capacity of CCD methods, including an empirical literature demonstrating that these methods can accurately detect true causes within observational data sets, when true causes were previously known: and a literature providing rigorous mathematical proofs of these methods for causal inference^1,2,11–16^. CCD methods have yielded important advances in non-psychiatric medical fields and have been successfully applied in psychiatric research, to large extent by our group^17–27^. Although there are strong reasons to believe that application of CCD methods to psychiatric research can lead to similar advances, it must be acknowledged that these methods have rarely been applied to psychiatric data. Accordingly, findings yielded from research employing CCD methods in psychiatry should be appraised with a commensurate level of scrutiny. The present study is among the first to apply CCD methods to discover causal factors for PTS. Next, we provide a brief, non-technical description of CCD methods for readers who may be unfamiliar with the literature upon which they are based. A more detailed description is provided in Supplementary Material 1.

## Causal inference from observational data

The following definitions of direct and indirect causation is explicitly or implicitly used by all state-of-the-art CCD algorithms and is consistent with what is considered to be causal in biomedicine (“Defining direct and indirect causation” subsection). The capacity of these methods to infer the causal processes generating the data that is observed (“Mapping of data to the causal...” subsection), follow from these definitions, as will be described^1,2^.

### Defining direct and indirect causation

#### Definition 1 (operational criterion for causation)

Assume that a variable A can be manipulated by a hypothetical experimenter to take values, each one denoted as a_i_. Assume also that the experimenter can manipulate A (e.g., give a drug to a patient or not, where A stands for type of treatment received). We denote the manipulation of A by the experimenter to take value a_j_, as: do(A = a_j_). If the experimenter assigns values to A according to a uniformly random distribution over values of A, and then observes P(B|do(A = a_i_)) ≠ P(B|do(A = a_j_)) for some i and j, (and within a time window dt), then variable A is a cause of variable B (within dt). Intuitively, P(B|do(A = a_i_)) ≠ P(B|do(A = a_j_)) means that manipulating A (i.e., do(A = a_i_) or do(A = a_j_)) results in a different distribution of variable *B* in the manipulated population.

#### Definition 2 (direct and indirect causation)

Assume that a variable A is a cause of variable B according to the operational criterion for causation in definition 1. A is an indirect cause for B with respect to a set of variables V, if and only if A is not a cause of B for some assignment (by manipulation) of values of V – {A, B}, otherwise A is a direct cause of B. Variables representing direct causes serve to mediate the relationship between an indirect cause and the effect.

### Mapping of data to the causal process generating the data (i.e., inferring causes from observational data)

Notice that the above definition hinges on experimental data. To be able to infer causality from observation data, we need to map the observation data to the causal process that generates the data. The existence of a set of causal relationships of the type “A causes B” in the vast majority of distributions (so-called “Faithful” distributions) guarantee that specific conditional dependencies and independencies will be observed in the data in a way that directly corresponds to causal mechanisms of the process that generates the data. For example, consider the situation that A directly causes B, B directly causes C, and no other direct causal relationship exists among them, and that the distribution is Faithful. In this case, all variables are mutually correlated (or broadly speaking dependent, if correlation is not the most appropriate form of measured association). Also, A will become independent of C if we condition on B (i.e., knowing the values of B blocks the association/dependency/information transfer between A and C). This is an application of the Causal Markov condition (CMC), a distributional assumption that allows us to infer all statistical independencies observed in data if we know the causal process generating the data. The distributional assumption of the Causal faithfulness condition (CFC) says that all independencies in the data are represented by the causal graph representing the causal process combined with the CMC. If the CMC and the CFC hold, it follows that all dependencies and independencies in the data correspond precisely to (i.e., form a perfect map of) the true causal structure that generated the data. In other words, an algorithm (or analyst if the number of variables is really small) can measure dependencies and independencies in the data and correctly infer the existence or non-existence of precise direct and indirect causality without conducting experiments. The Combination of CMC + CFC constitutes the aforementioned distribution assumption of Faithfulness. The presence of non-faithful distributions can undermine the accuracy of causal inference. Fortunately, it is known that among all possible distributions, non-faithful distributions are exceedingly rare (Lebesque measure 0)^28^ and of the few cases where faithfulness is violated, special algorithms are available to help manage this problem^29^.

### Discovering the most parsimonious predictive and simultaneously local causal relations in the data

A particularly powerful discovery tool—and one centrally related to methods we apply—concerns the Markov boundary of a target outcome variable. The Markov boundary provides the smallest set of variables that achieves maximum possible predictive accuracy of the target variable (most parsimonious prediction) under broad assumptions about classifiers used, data distribution, and error metrics of interest. The Markov boundary not only enables most parsimonious prediction, it also possesses a causal function since, in the majority of distributions, and when confounding is restricted, it includes the direct causes, direct effects and direct causes of the direct effects of the target variable^1,2,11–13^.

### Terminology

The previous discussion—following the Computational Causal Discovery literature—uses the term “cause” or “causal factor” to connote any variable that provides the potential to change the distribution of an outcome variable, should the distribution of the “causal” variable be changed. In contrast, change in distribution of an outcome from change in distribution of a non-causal correlate or predictor of the outcome, is not possible. Since our study is not designed to test actual change in distribution of outcome from change in causal factor, such factors may be more accurately described as “putative causes” or “putative causal factors” and such a qualification should be understood whenever we use the term “cause” or “causal factor” in this article.

## Causality and disorders of complex etiology

The accurate identification of direct and indirect causal factors for outcomes of interest provides the potential to reveal novel intervention approaches, targeting the identified putative causal factors. The utility of this knowledge may, however, be limited when the outcome studied has a complex etiology (almost certainly true of PTSD and many other psychiatric disorders)^25,30–33^. For such disorders, it is expected that there will be a very large number of interacting causal pathways (of linked indirect and direct causes) to influence the expression of the disorder. Thus, targeting only a small proportion of these causal factors/pathways may not result in successful intervention on the disorder (as many causal factors will remain to influence the disorder). However, knowledge about the complexity of a disorder may reveal unique opportunities for intervention. In a complex system, a small number of causal factors will influence a large proportion of causal paths, and most causal factors will influence a small proportion of paths^34–37^. We integrate methods designed to leverage this opportunity by detecting those putative causal variables with influence over the greatest proportion of paths into the target outcome variable. We emphasize that contrary to methods designed to discover specific direct and indirect causes to a target outcome variable—that provide guarantees for correctness under well specified conditions—network connectivity-based approaches for determining putative causal variables with broadest influence are presently a heuristic strategy. This heuristic however has the advantage that it describes higher-level properties than methods designed to only infer direct and indirect putative causes.

In the research described next, we apply a specific protocol—the *Protocol for Computational Causal Discovery in Psychiatry (PCCDP)*—to implement the causal discovery methods just described, to observational data sets used in the field. This protocol was applied to discover a complex network revealing the causes and effects of PTS in police officers using a particularly compelling observational data set for this purpose^38,39^. This data set contains prospective information on a diversity of risk variables collected prior to critical incident exposure, from the peritraumatic period during the first year of police service, and in the post trauma period, one year after the start of police service. Several important findings on risk for PTS were published from this data set including relations between pre-trauma salivary 3-methoxy-4-hydroxyphenylglycom (MHPG) responses to experimental stress challenge during training and peritraumatic dissociation and PTS symptoms during police service^40^, between cortisol awakening response during training and peritraumatic dissociation and acute stress responses during police service^41^, and between workplace stress^42^, fear-potentiated acoustic startle^43^, positive and negative emotions during training^44^, cortisol responses to experimental stress challenge during academy training^45^, killing or seriously injuring another person in the line of duty^46^, trait anger^47^, alexithymia^48^, and gender and ethnicity^49^ with PTS symptoms. None of these findings were based on methods that could enable causal inference. Of note: PCCDP is an updated version of a previously published protocol called the Complex Systems-Causal Network (CS-CN) method^25^.

## Methods

### Participants

The NYU/UCSF Police Prospective Longitudinal Study (NYU/UCSF-PPLS) was conducted by Marmar and colleagues in a study to understand risk for PTS in a prospective longitudinal cohort of police academy recruits from four police departments (New York City, San Francisco, Oakland, San Jose)^38,39^. All police academy recruits were eligible for inclusion in the study, except those who had previously served in the military, law enforcement, or emergency services. Informed consent was obtained from all subjects. Procedures were approved by NYU Medical Center and University of California, San Francisco, Institutional Review Boards.

Details on the procedure used in the collection of data and on the variables measured can be found in Supplementary material 2, including Supplementary Table S1 indicating measurement of genes. To briefly summarize this procedure: on entry to police academy training, subjects were recruited and following informed consent, a baseline assessment battery of self-report measures was administered in person or by mail to subjects, consisting of measures of variables hypothesized to indicate risk for PTS from prior to Police Academy training and of risk variables on symptom expression and functional status related to adjustment to the police force. At 12-month after completion of Police Academy training, this assessment battery was repeated, excluding those scales measuring pre-police academy risk. This 12-month follow-up assessment also included measures of critical incident trauma exposure, peritraumatic responses to such critical incidents, and assessments of the outcomes of PTS and depression. During police academy training, and after baseline assessments were completed, subjects completed psychophysiologic and neuroendocrine assessments, and genetic testing.

Because this study is focused on the etiology of PTS, data on a sub-sample of officers (*n* = 207) who were exposed to at least 1 duty-related life-threatening event in the first 12 months of police service was used (from the larger sample of police academy recruits, *n* = 400). Demographic differences between subjects in our sub-sample and the larger sample were compared. No differences were found on the demographic characteristics of age, gender, education, or marital status. However, differences were found on ethnicity (*χ*^2^ = 7.2, *P* = 0.007 two-sided), with the proportion of “Caucasian” being lower in the full sample (38% vs. 43%). The difference in ethnicity would not be expected to confound any observed associations since the conditional independency tests within our methods—detailed below—would first exclude ethnicity (and all other possible measured confounders) before concluding that any causal relation is present in the data.

Collection of genetic data began after the study was initiated, and thus genetic data were measured for a selected sub-sample of subjects (*n* = 157). Hence, the etiological relationship among genetic and behavioral measures can only be determined for this sub-sample.

## Procedure

**Fig. 1:**
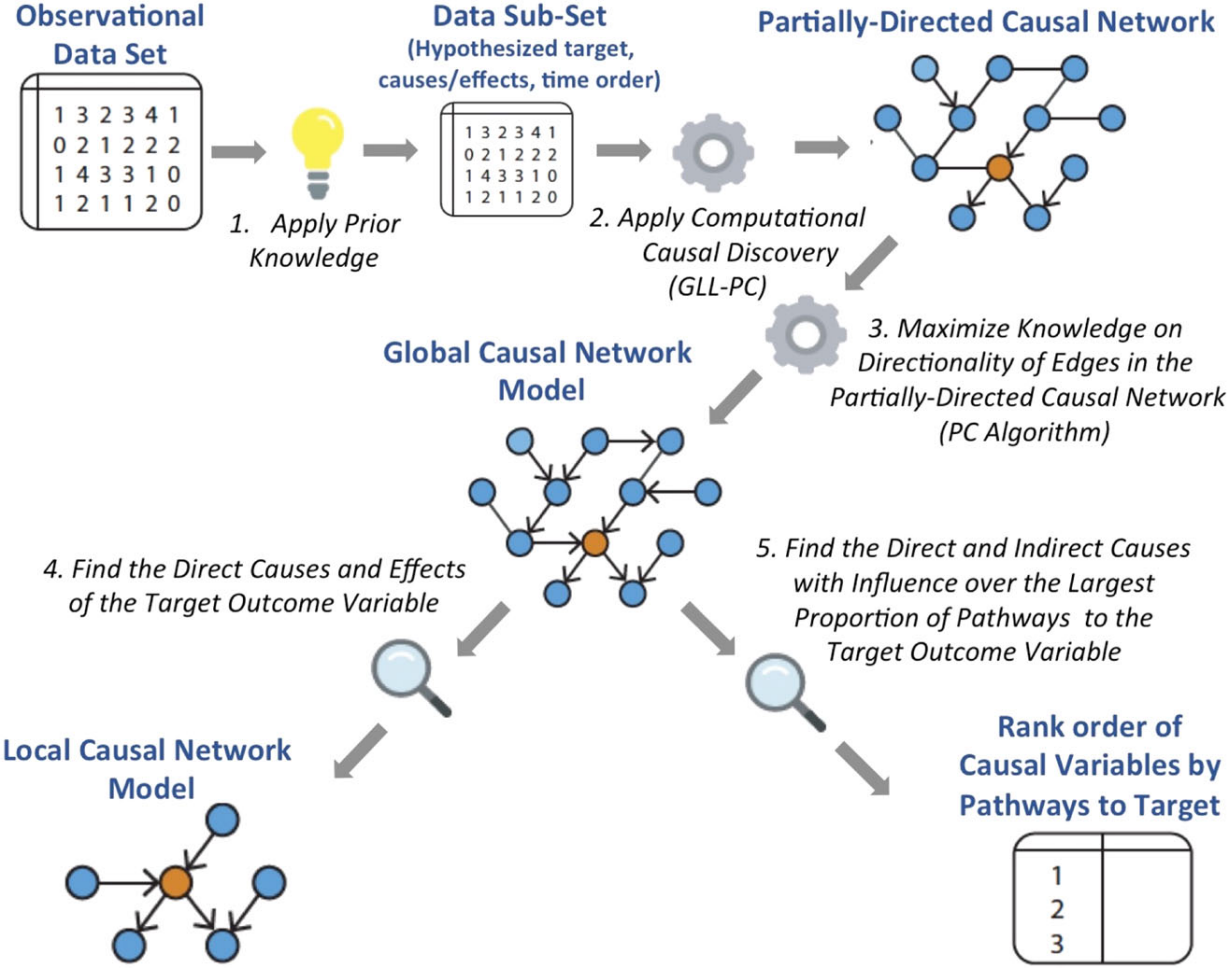
The Protocol for Computational Causal Discovery in Psychiatry (PCCDP). Procedures used to discover causes, from the NYU/UCSF Police Prospective Longitudinal Study data set.

PCCDP follows a specific set of steps that integrates an investigator’s prior knowledge in the process of CCD, to discover specific causes and effects of psychiatric disorders of interest. These steps are described next and illustrated in Fig. 1.

### Apply prior knowledge to an observational data set to create a data subset based on hypothesized causes and effects of a designated target outcome variable

As illustrated in Fig. 1, PCCDP allows investigators to apply their prior knowledge about the phenomenon under study to identify variables and relations considered for analysis. Broadly, PCCDP considers two types of prior knowledge.

#### Prior knowledge on the variables to include in the analysis

Although, an important advantage of the algorithms used within PCCDP is their capacity to consider large numbers of variables in samples of modest size, thus minimizing the considerable risk of missing direct and indirect causes, and failing to analyze hidden common causes that would confound the results of causal discovery. It is also advantageous to be guided by the literature in one’s field, to exclude variables from consideration: (a) when there is no reason to believe the variable would have any relation to the target (e.g., For PTS target: subject’s color preference, brand of subject’s personal motor vehicle), (b) when multiple variables measure the same construct (e.g., a data set contains four similar variables measuring depression). This first application of prior knowledge (i.e., variable consideration) seeks to constrain the introduction of superfluous information into the process of causal modeling, and to minimize potential violations of faithfulness.

#### Prior knowledge on the relationship between variables

Prior knowledge on the relationship between variables, such as (i) time order between variables based on knowledge of when a variable was measured or would have exerted its effect or, (ii) causal relationships that have been established from prior experimentation. This second application of prior knowledge seeks to leverage known facts about relationships, in the process of causal modeling (e.g., fact that causal relations cannot go backwards in time; fact that experiments can determine causal relations).

In the present study, prior knowledge was applied by searching the NYU/UCSF-PPLS data set to select variables/relations for inclusion in a data subset for computational analysis, as follows:

1. *Selection of the outcome target*: The NYU/UCSF-PPLS data set was reviewed for variables that best measured PTS in a sufficient number of subjects. The primary target variable used in this study is PTS Symptom Severity (PTS Sev) as measured by the total score of the PTSD Checklist (PCL) at 12 months of Police Duty^50^.
2. *Selection of hypothesized cause and effect variables*: One hundred and forty-eight variables were pre-selected based on their plausibility of representing causes or effects of PTS Sev, and absence of information equivalency with included variables. Such variable pre-selection was guided by prominent theory in the field^30–33^. Variables were chosen to cover a diversity of modalities including genetic, physiologic, neuroendocrine, developmental, social, family, emotional, behavioral, and cognitive variables. Details on the measurement of the 148 variables in the data set are provided in Supplementary material 2.
3. *Designation of time époques for measured variables*: The temporal order of variables designated for the present analysis was based on the order in which they were believed to exert their influence. In the present study variables were categorized into seven time époques: (1) constitutional (demographic and family history variables collected on entry to Police Academy), and genetic variables assessed during Police Academy training, (2) childhood (variables collected on entry to the Police Academy, concerning subject’s childhood), (3) entry to Police Academy (i.e., variables collected on entry to the Police Academy about subject’s symptoms and functioning related to adjustment to the Police Academy), (4) during Police Academy training (psychophysiological and neuroendocrine variables measured after baseline self-report assessment was completed, but before the end of Police Academy training), (5) critical incident exposure during the first 12 months of police service (measured at 12-month follow-up), (6) peritraumatic period (i.e., self-reports of responses to duty-related critical incidents during the first 12 months of police service, measured at 12-month follow-up), (7) post 12 months of Police service following completion of Police Academy training (self-reports of symptoms and functioning, including depression and PTS, measured at 12-month follow-up).

The resulting data subset was then input for computational processing, along with a defined variable table, describing each variable, its numeric type (e.g., ordinal, cardinal) and its time époque. Importantly, no discretion over the processing of variables was allowed, once the data subset and variable table was entered as input. Each variable in the data subset can be found as in the original NYU/UCSF-PPLS data set, variables were also renamed for the sake of clarity and graph visualization. The list of variables and their corresponding original names and designated time époques can be found in Supplementary material 3.

### Apply computational causal discovery methods to the data subset to determine a partially directed causal network

Causal edge detection is accomplished by integrating a well-validated set of algorithms called GLL-PC^11,12^, which discover causal edges by using tests of conditional independence among variables in the data subset with the semantics/definition of a causal edge offered in the Introduction^1,2^. GLL-PC employs local causal discovery and is followed by local-to-global algorithms from the best-of-class local to global learning (LGL) families^1,2,11–14^. As shown in Fig. 1, GLL-PC processes the data subset to create a causal network via the inference of all causal relationships consistent with the data. In addition to identifying putative causal relationships, the inferred network can also be used for optimal predictive modeling via elicitation of the Markov boundary for each of the variables. As described, the Markov boundary of a given variable, provides the smallest set of measured variables that achieves maximum possible predictive accuracy and is also most parsimonious under broad assumptions about classifiers used, data distribution, and error metrics of interest^11–13^. Because GLL-PC is provided with time époque information, it is also able to provide direction between many of the variables. Within PCCDP, GLL-PC is programmed to draw direction from variables forward in time (when time order is known). This procedure yields a partially directed causal network of nodes (variables) and the edges (bivariate causal relations) that connect them. For this study, GLL-PC was applied with the following fixed parameters: a *max-k* (maximum conditioning set size) of 3, and a *p*-value threshold of 0.05.

### Maximize knowledge on directionality of causal edges to determine the Global Causal Network Model of PTS

To orient any remaining undirected edges (i.e., edges between variables from same time epoque), PCCDP applies techniques derived from the PC algorithm^2^. Using “sepsets” from the conditional independence tests performed by GLL-PC, PC’s edge orientation rules are employed to orient Y-structures (e.g., A → C ← B) and propagate orientation of appropriate edges in the given undirected or partially directed causal network. Observational data can only resolve causal directions up to the Markov equivalence class, i.e., the set of causal graphs that share the same edge orientations consistent with the data, leaving the rest as unknown^2^; this procedure results in the Global Causal Network Model of PTS (Fig. 1).

### Find the direct causes and effects of the target outcome variable to determine the Local Causal Network Model of PTS

The previous computational steps create a Global Causal Network Model based on observed putative causal influences between all variables in the data subset. This Global Causal Network Model contains variables within what is called the local causal neighborhood of the target outcome variable (e.g., PTS Sev), and also the local causal neighborhoods of all variables in the data subset (in order to learn pathways of sequences between indirect and direct causes to the target outcome variable). PCCDP also reveals the Markov boundary for each variable since the latter comprises the direct causes, direct effects, and the direct causes of the direct effects of each variable, including PTS Sev. This Local Causal Network Model for PTS Sev is one of the two main “outputs” of PCCDP (Fig. 1).

### Find the direct and indirect causes with influence over the largest proportion of pathways to PTS

In general, once the global causal graph is discovered, a variety of graph search algorithms combined with quantitative causal inference can be used to derive sets of variables that separate a cause of PTS Sev from PTS Sev or that achieve a desired degree of influence on PTS Sev. Such calculations are very expensive and even prohibitive for large and complex networks, however. In the present work, we instantiate the PCCDP protocol with a heuristic strategy as follows: in order to determine the putative causal variables with influence over the largest proportion of pathways to the target, a subnetwork of all pathways in the Global Causal Network was examined that terminate at the designated target variable, restricted to four path-lengths or less (to exclude variables with distal influence on the target). PCCDP evaluates (heuristically) the effect of intervention on causal variables by determining the number of intact pathways to the target that become eliminated, when each putative causal variable is deleted. PCCDP produces, as output, a rank order of causal variables by the proportion of pathways to the target variable eliminated, when specific causal variables are deleted (Fig. 1).

#### Additional safeguards

##### Determining stability of causal edges through bootstrapping procedures

PCCDP integrates a bootstrapping analysis to determine the stability of the resulting Global Causal Network Model with respect to sampling variability. The data subset was bootstrapped 100 times and Global Causal Network Models were generated for each iteration. The stability of an individual edge was calculated as the percent (%) of bootstrapped networks in which it was detected.

## Software and tools

PCCDP was programmed in MATLAB R2016a^51^. Post-analysis network visualization was conducted in Cytoscape 3.6.1.^52^. Network pathway analysis was conducted in part using the Cytoscape plugin, Pathlinker^53^.

## Results

A Global Causal Network Model of 146 nodes and 345 edges was found (two nodes were disconnected from the network). Of the 345 identified edges, 125 were oriented based on time époque, and 201—from the same time époque—were oriented by the edge orientation procedures of the PC Algorithm. Thus, 326 edges (94%) were oriented by the PCCDP method and 19 edges (6%) remained unoriented. The complete edge list and edge stability estimated from the bootstrapping analysis, is provided in Supplementary material 4.

### Direct causes and effects of PTS (Local Causal Network Model)

**Fig. 2:**
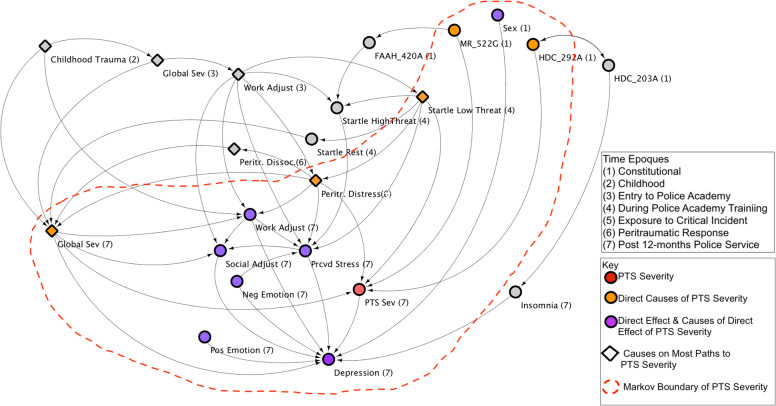
The second-degree Neighborhood of PTS Sev, including its Local Causal Network Model/Markov boundary. Putative causes and effects within two “steps” of PTS Sev.

In Fig. 2, we present a visualization of all second-degree neighbors of the PTS Sev variable (i.e., variables within two “steps” from PTS Sev), including a subset of these variables that are defined by the Local Causal Network Model (i.e., the Markov boundary of PTS Sev, represented by the red dotted line in Fig. 2). These Markov boundary variables reflect the minimal set of information required to predict the value of the target as accurately as the whole of data support this prediction. Conditioned on these nodes, PTS Sev is rendered independent of the rest of the nodes in the network. Figure 2 shows the direct causes, direct effects, and direct causes of the direct effects of PTS Sev (i.e., it’s Markov boundary). This network is configured to a hierarchical layout to best visualize the flow of causal influence.

This visualization is informative because it includes variables within the Local Causal Network Model and variables immediately “upstream” and “downstream” from many of these variables. The numeral in parenthesis within each node indicates the time époque (1–6) of the variable represented by the node. To make this visualization as clear as possible, we removed 6 nodes that were not on any causal pathway to PTS Sev—or within its Markov boundary. The PTS Sev node is colored red. Nodes colored orange represent the five direct putative causal variables to PTS Sev. Nodes colored purple represent either the one direct effect of PTS Sev (depression at 12 months) or the direct causes of this one direct effect. The red dashed line represents the Markov boundary of PTS Sev variable and, accordingly contains all its direct causes (orange nodes), its direct effects (purple nodes) and the direct causes of its direct effects (purple nodes). Nodes represented in diamond shape indicate variables with influence on the greatest proportion of causal paths to PTS Sev within the entire Global Causal Network that were also included in the set of nodes (Fig. 2). More detail on these nodes are described next.

### Direct and indirect causes on the largest proportion of pathways to PTS

**Fig. 3:**
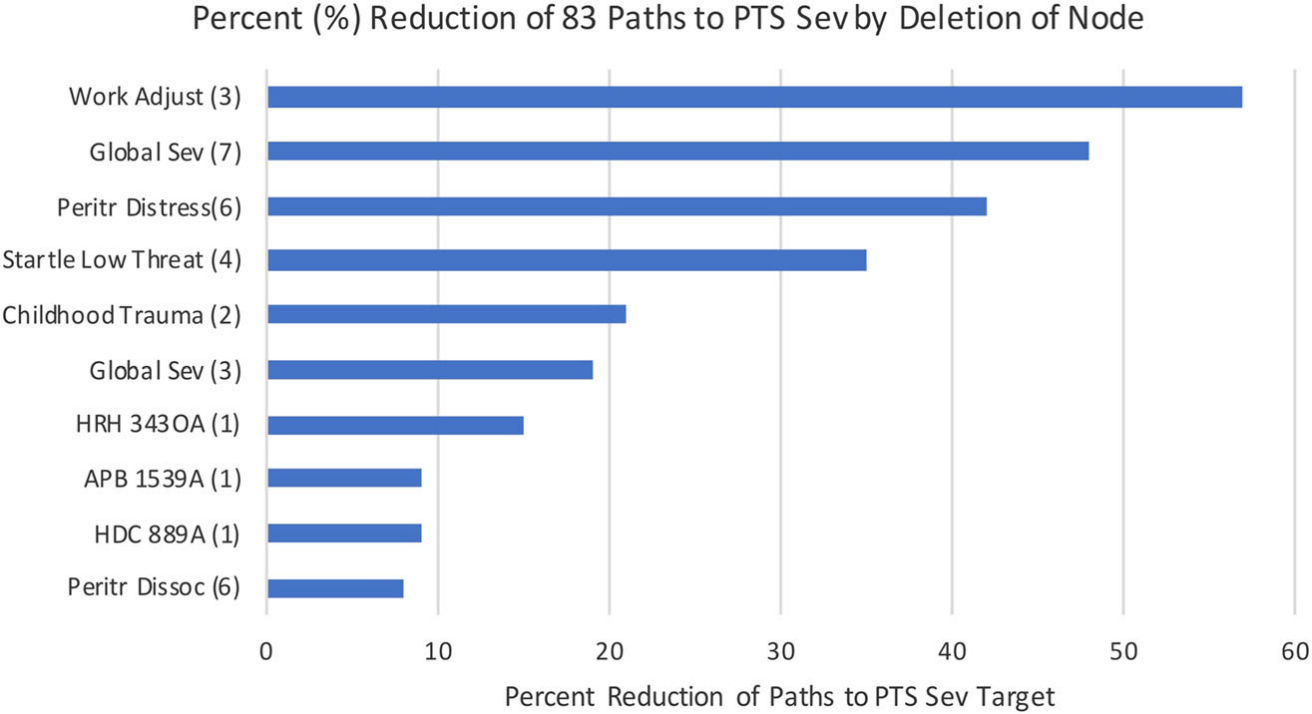
Rank order of variables with influence on greatest proportion of pathways to PTS Sev (time époque in parenthesis). Putative causes participating in the greatest proportion of the 83 pathways to PTS Sev (within four “steps” or less), from the Global Causal Network Model.

Searching the entire Global Causal Network Model for all paths of four steps or less to PTS Sev, yielded 83 unique paths. Modeling removal of nodes to determine those with influence on the greatest proportion of these 83 pathways, revealed 10 variables with such influence. Many nodes are part of multiple paths. We heuristically evaluate the effect of intervention on each node by computing the proportion of paths eliminated when a node is deleted from the network. In Fig. 3, a rank order of these 10 variables along with the proportion of the 83 pathways that a given variable was found to influence, are provided. Seven of these 10 variables were contained within the second-degree neighborhood of PTS Sev (represented by diamond shape in Fig. 2). The remaining three variables lie outside the second-degree neighborhood and, accordingly, are not shown in Fig. 2. A list of the 83 pathways to PTS Sev is provided in Supplementary material 5.

## Discussion

The overarching aim of our study was to apply computational causal discovery methods to clarify the complex etiology of PTS, in order to identify promising new approaches for intervention. As detailed in the “Introduction” section, knowledge appropriate for intervention must be based on causal inference, and such inference cannot be made with confidence from research using observational, correlational methods. Since human etiological experiments on PTS can so rarely be conducted, we sought new methods to infer causes to inform intervention. How may our results inform intervention on PTS?

Our results revealed a network that includes five direct putative causal factors for PTS: SNPs for the *Histidine Decarboxylase (HDC)* and *Mineralocorticoid Receptor (MR)* genes, startle low threat (acoustic startle response in a low perceived threat condition during academy training), peritraumatic distress to critical incident exposure during the first year of police service, and general symptom severity at 12 months. These five direct causal factors are influenced by a wide variety of indirect causal factors on 83 pathways to PTS. Of the five direct causal factors, three were found to influence a large proportion of the 83 pathways: general symptom severity at 12 months (47% of paths), peritraumatic distress (42% of paths), and startle low threat (35% of paths). Several indirect putative causal variables were also found to influence a large proportion of causal pathways, some influencing a greater proportion of pathways than any direct cause. For example, work adjustment on entry to police academy training influenced 57% paths, childhood trauma influenced 22% of paths, and general symptom severity on entry to training influenced 18% of paths.

Regarding the relevance of this information for revealing promising intervention targets: optimally, interventions would be available that can modify each of the five direct putative causes, as they would thereby separate PTS Sev from all its sources of causal influence (since all indirect causes must exert their effect through at least one direct causal factor). One cannot presume, however, that all five causes are modifiable: and some—though modifiable—may have already exerted their effect on likelihood of PTS at the time an intervention is considered (e.g., when delivered after the peritraumatic period, an intervention designed to modify peritraumatic distress would not be expected to change the likelihood of PTS). Therefore, knowledge on indirect putative causes—especially those that participate in a large proportion of causal paths—may provide useful knowledge on promising intervention targets, and complement knowledge on direct causes. As described in the method section, our approach to integrating knowledge on indirect causes, by examining the proportion of causal paths in which they participate, is heuristically derived. Ultimately, knowledge to inform intervention will require causal effect estimation methods to determine unit change in outcome variable per simulated unit change in (direct or indirect) causal variable. Such methods, based on Pearl’s “Do Calculus”^1,15^, have recently become available and offer promising avenues for future research.

Examining the putative causes shown in Fig. 2 reveal several biologically significant pathways to inform understandings on the pathophysiology of PTS and reveal promising intervention targets. The *HDC* gene, for example, indicates a role for the histamine system. There is evidence that histamines are involved in the regulation of processes thought to be central to PTS including arousal, sleep, inflammation, and central nervous system response to provocation, including regulation of the hypothalamic–pituitary–adrenal (HPA)-axis^54,55^. Consistent with the results of a previously published study^56^, our findings indicate that widely available antihistamine agents may have promise for intervening on PTS. As another example of a biologically significant result, with corresponding intervention potential: the *MR* gene codes for expression of the mineralocorticoid receptor. MR receptors have a role in regulating the release of norepinephrine in response to stress, and this release is thought to be responsible for the consolidation of traumatic memory^31,57–59^. Agents that block the activity of this system have been demonstrated to be efficacious for PTS^60,61^.

Our findings on fear-potentiated startle reactions may have particular relevance. Neurobiological systems responsible for startle have been implicated in the etiology of PTS for decades^62–64^. Startle responses are necessary for survival: quickly orienting the brain towards sources of threat, and preparing the body to quickly react to these perceived threats with survival-preserving behavior. The heightened startle responses and poor startle habituation commonly found in individuals with PTS have been described as indicators of dysfunctional fear learning, contextual processing, and attentional biases towards threat^31,33,43,57,65^.

It is noteworthy—as shown in Fig. 2 that startle reactions, especially startle low threat, have broad “downstream” influence on other variables leading to PTS and depression. These “downstream” variables include peritraumatic distress, perceived stress, and global symptom severity: and, indirectly, work adjustment and social adjustment. Startle reactions in conditions of low threat would indicate a neurobiological system primed to respond with survival-preserving behavior to stimuli that are not objectively threatening, and such a system would be guided by attentional biases towards threat, described in previous research^65^. Attentional biases toward threat can be highly maladaptive, leading to misperception of neutral cues as threatening and limiting the capacity to extinguish the fear response to such cues, once learning occurs. Such attentional biases would be expected lead to heightened levels of distress in the peritraumatic period, and continuing levels of perceived stress, mental health symptom severity, and ongoing difficulties in social and work adjustment: consistent with the results shown in Fig. 2.

As shown in Fig. 2, startle low threat and startle high threat are influenced by “upstream” variables of history of childhood trauma and previous psychopathology. Animal studies have reported heightened startle responses and poor startle habituation following repeated prior stressors^66,67^ and our findings may indicate—consistent with these previous studies—that the startle responses we observed, were potentiated by repeated prior stress exposures. Another interesting source of “upstream” influence on startle is indicated by the path from the *MR gene* to Fatty Acid Amine Hydrolase (*FAAH) gene* to startle high threat. Such a path may have biological significance as the endocannabinoid system has recently been implicated in PTS and has strong regulatory relations to the sympathetic nervous system^68,69^. The *FAAH* gene encodes a protein that hydrolyzes the endogenous cannabinoids anandamide and 2-arachidonoylglycerol (2-AG)^70^. Our findings highlight an important putative causal relation between the endocannabinoid system, the sympathetic nervous systems, startle, and PTS.

Our study offers unusual opportunities for untangling complex causal relations and for yielding knowledge on preventative intervention, as the data set included many risk variables measured prior to trauma exposure. The vast majority of studies on startle reactions and PTS, for example, measured startle in subjects who were already trauma exposed or who had already acquired PTS. In the present study, startle was measured during police academy training, and prior to any reference critical incident related to police duty. Our findings indicate that the observed startle responses are putative causal factors—not effects —of PTS, and thus convey opportunities to intervene on PTS via intervention on startle. Police officer candidates who display the patterns of startle response observed in this study may be good candidates for preventative intervention, including administration of agents that serve to attenuate the startle response^71,72^.

Another result with promise for preventative intervention—given its pre-trauma measurement—is work adjustment during police academy training. Work adjustment is relatively easy to assess and—should such a problem be revealed during academy training—police officers could be offered more support in adjusting to work, in the service of reducing their likelihood of developing PTS during police duty. Work adjustment is based on police officer reports of feeling inadequate at work, impaired work performance, and days absent from work around their entry to police duty.

Our findings can also help clarify the nature of the well-described comorbidity between PTS and depression^73^, which has also been reported in subjects from the sample used in the present study^74^. Do symptoms of PTS and depression emerge and persist independently; or do they influence each-others’ expression? If there is dependence between them: what is its direction? Our findings indicate that post-traumatic depression and PTS are related: PTS is causal of depression; depression is not causal of PTS. Our methods were able to determine directionality in this context, by using the sound edge orientation procedure of the PC algorithm. The capacity to determine the directionality of relationships is a particularly important implication of our research. Our results indicate that intervention on Depression cannot improve PTS, but intervention on PTS can improve depression. Of course, comorbid depression in a police officer should be treated, but this treatment should not be expected to improve PTS.

As described, although these results are intriguing, and may eventually serve to advance the field, they were determined using methods that have rarely been applied in psychiatry, and so caution must be used in accepting their veracity. Future experimental research—where possible—may be used to confirm results and—where not possible—it will be important to replicate results using similar methods with other observational data sets. Additionally, PCCDP cannot (at its present configuration), rule out the possibility that an unmeasured hidden common cause may explain an observed causal relationship in our model. At present, the methods employed enable the consideration of far more variables (146) than can possibly be considered using conventional methods and common causes from these 146 measured variables can safely be excluded. Our analyses do not, however, rule out the possibility of confounding from unmeasured variables. It is in principle possible to detect such effects by using additional methodologies^2,75^, and this is an avenue for future work. As described in the method section, genetic data was only collected in a sub-sample of subjects (*n* = 157) and therefore our findings on the relationship between genes and behavioral measures are only relevant for this sub-sample. Our methods also did not use a diagnostic measure of PTSD, but rather used a PTS symptom severity measure. Future research would benefit from such diagnostic measures. We also hope that the results presented may contribute to knowledge on diagnostic classification related to trauma responses. Finally, although our methods include highly rigorous processes to support the generalizability of the resulting models, our resulting models were not validated with an independent sample of police officers. Should suitable data sets become available, the generalizability of our findings would be supported through validation with such independent samples.

## Conclusions

A diversity of variables—some representing processes that are readily remediable—were identified as direct causes of PTS in police officers. Other remediable causal variables were found to influence a large proportion of the causal paths. Knowledge of the set of direct putative causes—and the putative causes with broad influence over pathways—provide investigators with unique and valuable knowledge to consider novel intervention strategies. Obviously, experimental research would be very important in the pursuit of such knowledge but, as detailed previously, such research can rarely be conducted with humans. Thus, the application of CCD methods may offer new avenues to advance intervention on PTS.

## Supplementary information

Supplement 1

Supplement 2

Supplementary 3

Supplementary 4

Supplementary 5

## Data Availability

All algorithms used have been previously published in full detail in the peer reviewed literature and referenced in this article. They are free to use for scientific, non-commercial use. Therefore, all results in this article can be reproduced exactly by knowledgeable practitioners (i.e., machine learning scientists who can competently code algorithms). The specific implementation used in the paper is proprietary (licensed by Discovery Holdings LLC to the Aliferis Lab).
