# Supplementary material for: Computational causal discovery for post-traumatic stress in police officers
Source: Transl Psychiatry. 2020 Aug 11;10:233. doi: 10.1038/s41398-020-00910-6 (PMC7417525; doi:10.1038/s41398-020-00910-6)
Supplement: Supplementary file 2 — Supplement 2 [file 41398_2020_910_MOESM2_ESM.docx]

Supplement 2

**Measurement of Variables in the Police Recruit Data Set**

1. *Participants and Procedures*: Officers from four police departments (New York City, San Francisco, Oakland, San Jose) were recruited into a prospective study of bio-psycho-social predictors of stress responses to critical incident exposure. Trainees who had previously served in the military, law enforcement, or emergency services were excluded. Procedures were approved by the University of California, San Francisco and New York University Langone Medical Center Institutional Review Boards. Participants were evaluated on a comprehensive set of clinical, self-report, neuroendocrine and physiological measures during academy training and then were re-evaluated on self-report measures at 12-month reassessment. Because this study is focused on the etiology of PTSD, data on a subsample of officers (n = 207) who were exposed to at least 1 duty-related life-threatening event in the first 12 months of police service were used (from the larger sample of police academy recruits, n = 400) (for a full description of recruitment procedures see^1^).
2. *Baseline Assessment* included a battery of self-report measures administered in person or by mail at entry to police academy training consisting of measures of variables hypothesized to indicate risk for PTS from prior to police academy training (e.g. history of childhood trauma, and family history of mental health or substance abuse) and variables concerning symptom expression and functional status related to adjustment to the police force, hypothesized to indicate risk for PTS (e.g. work adjustment, marital adjustment, negative emotion).
3. *12-month Follow-up Assessment* repeated the same Baseline Assessment measures, excluding those measuring pre-police academy training risk, at 12 months of police duty. This 12-month Follow-up Assessment also included measures of Critical Incident Trauma Exposure, Peritraumatic responses to such critical incidents, and assessments of the outcomes of PTS and Depression.
4. *Additional Assessments*: During Police Academy training (and after Baseline Assessments were completed), subjects completed assessments of psychophysiologic and neuroendocrine responses, and genetic testing.

**Measures**

# Baseline Assessment comprised a battery of self-report measures administered in person or by mail at entry to police academy training. The measures (and the variables they measured), were:

# The Family History Screen (FHS): The FHS^2^ is a 28-item measure used to collect psychiatric information on a patient’s first-degree relatives, was administered in order to assess *family history of substance abuse, family history of mood and anxiety disorders*

1. **Dissociation Experience Scale (DES):** The DES^3^ is a 28-item measure that asks people to describe the degree/percentage (0-100) of time in their daily lives in which they experience memory lapses and other forms of dissociation. *Dissociative symptoms*

# Social Adjustment Scale-Self Report (SAS-SR): The SAS-SR^4^ is a 54-item measure of social adjustment that has been validated on clinical and non-clinical populations. Subscales of *Marital Adjustment*, *Social Adjustment, and Work Adjustment* were utilized in the current study.

1. **Positive and Negative Affect Schedule (PANAS)***.* Positive and negative emotions were assessed during academy training using the PANAS.^5^ Participants rated the extent to which they experienced 20 emotion items on a 5-point Likert-type scale ranging from 1 (“*very slightly or not at all”*) to 5 (“*extremely”*) over the past year. The mean of 10 items comprise the Positive Activation (PA) subscale and 10 items comprising the Negative Activation (NA) subscale. These scales were used to measure *Positive Emotion* and *Negative Emotion*, respectively.
2. **Hopkins Symptom Checklist 90-R Global Severity Index (SCL-90-R GSI)***:* Self-reported distress from psychological symptoms was measured using a combination of 29 items from multiple scales of the Symptom Checklist-90-R that make up the Global Severity Index (GSI)^6^ which measures the variable of *Global Severity.*
3. **Early Childhood Trauma Inventory (ETI-SR):** The ETI-SR^7^ short form version is used to assess frequency, onset and impact of various forms of childhood abuse. This measure includes 27 yes-no items related to experiences of sexual, physical, emotional, and general abuse/trauma (i.e., natural disaster) before the age of 18**.** This instrument measure the variable of *Childhood Trauma.*
4. **Insomnia Severity Index (ISI):** The ISI^8^ is a 7-item measure on a 5-point likert-type scale used to assess sleep difficulty and diagnose insomnia, by inquiring about sleep disturbances as well as the extent to which sleep patterns are stressful/worrisome. The ISI was used to measure the variable of *Insomnia*.
5. **Pittsburgh Sleep Quality Index (PSQI):** The PSQI^9^ is a 19-item measure used to assess self-reported sleep quality and disturbances over the past month. The responses yield one global score for the variable of *Sleep Quality.*
6. **State Anxiety (STAXI-2):** The STAXI-2^10^ is a 10-item measure of *State* *Anxiety* and *State Anger* that uses a 4-point frequency scale (“almost never” to “almost always”).
7. **Sources of Support (SOS):** The SOS^11^ is a 10-item measure assessing perceived social support. Participants rate their emotional and instrumental support in a yes/no format. Higher total scores reflected stronger perceived *Social Support*.

12-month Follow up Assessment: The Baseline Assessment was repeated at 12-months of police duty, excluding measures of pre-police academy training risk factors (FHS, ETI-SR). Measures of Critical Incident Trauma Exposure, Peritraumatic responses, Posttraumatic Stress, and Depression were added to comprise the 12-month Follow-up Assessment battery of measures. These additional self-report measures were:

1. **Critical Incident History Questionnaire (CIHQ):** The Critical Incident History Questionnaire (CIHQ)^12^ is a 39-item self-report measure designed to assess exposure to PTSD criterion ‘A’ events typically encountered by police officers in the line of duty. In the current this variable was calculated related to the number of life-threatening events the officer was exposed to in the first 12 months of police duty. This variable is labeled as *Life Threat*.

# The Peritraumatic Dissociative Experiences Questionnaire (PDEQ): The PDEQ,^13^ a 10-item measure, was cued to participants self-identified most distressing traumatic events where they rate items on a 5-point Likert scale (from 1 = not at all true to 5 = extremely true). This variable is labeled as *Peritraumatic Dissociation*.

# The Peritraumatic Distress Inventory (PDI): The PDI^14^ is a 21-item measure cued to where participant’s self-identified most distressing and traumatic event in which participants rate items on a 5-point Likert scale (from 1 = not at all true to 5 = extremely true). This variable is labeled as *Peritraumatic Distress*.

1. **The Beck Depression Inventory** **Revised** **(BDI-II):** The BDI-II^15^ was used to assess depression symptoms at 12-month follow-up. The BDI-II is a 21-item (4-point scale) self-report instrument, designed to assess the severity of depression symptoms over the preceding week.
2. **The PTSD Checklist (PCL):** The PCL^16^ is the instrument used to measure the primary target variable used in this study, PTS Symptom Severity (PTS Sev) as measured by the total score of the PTSD Checklist (PCL) at 12 months of Police Duty.

Additional Measures: *Psychophysiological and Neuroendocrine Measures Administered During Police Academy Training*. The following procedures (and the variables measured) were conducted during police academy training, and after the Baseline Assessment was completed.

1. **Acoustic startle**: Acoustic startle responses to loud noises were assessed on entry to police duty. Three physiological response measures were obtained: skin conductance level, heart rate, and electromyogram (EMG) of the left eye blink reflex. Habituation slopes were calculated for *Skin Conductance*, *EMG,* and *Heart Rate*. Two cues indicated either the onset of a burst of white noise or a “fear potentiated” mild finger shock. Before and after each trial, participants completed ratings of subjectively felt fear in response to no threat (white noise) low threat, or high threat, and variables were created for *Startle at rest* (baseline) and *Startle Low Threat* and *Startle High Threat* to capture a subject’s differential response to threat signals.
2. **Critical Incident Video Challenge Test:** Participants observed an innocuous travelogue video for 10 minutes, followed by a 20-minute critical incident depiction video, then a 20-minute travelogue video again during the response period and changes in cortisol and 3-Methoxy-4-hydroxyphenylglycol (MHPG) were measured in response to the provocation. Saliva was collected at three time points; T1 (Baseline) was a baseline measure obtained immediately following the first travelogue video and just prior to the viewing the video stressor, T2 (Initial Cortisol/MHPG Response) was a measure obtained immediately after the conclusion of the 20-minute video stressor, and T3 (Final Cortisol/ MHPG Response) which was obtained at the conclusion of the 20-minute response period. Area Under the ROC (AUC) change across the time points was calculated for both cortisol and MHPG response to the challenge following and defines the variables *Cort Video Challenge* and *MHPG Video Challenge*.

# Cortisol Awakening Response and Low Dose Dexamethasone Suppression Test (DST): Saliva cortisol was collected to measure cortisol awakening response before and the next day after administration of 0.5 mg of dexamethasone at 11pm. Four saliva samples were collected at 1, 30, 45 and 60 min after awakening on both days. The salivary cortisol data were consolidated into three variables: (1) pre-dexamethasone or basal cortisol level (the AUC created by the four samples collected after awakening on day 1), (2) post-dexamethasone cortisol level (the AUC created by the four samples collected after awakening on day 2), and (3) the percentage of dexamethasone suppression of cortisol (pre-dexamethasone minus post-dexamethasone AUC/pre-dexamethasone AUC)×100.^17^ These variables are labeled as *Cort Awake* *Dex* and *Cort Awake No Dex*.

1. **Genetic Testing:** Genetics (n=157): Participants were given the option of completing a one-time blood draw for genetic testing. From 2008 to 2010 a sub-group of 157 officers, 107 New York participants and 50 Bay Area participants) consented to provide a 34 ml blood sample, drawn by a certified phlebotomist. In the current study, SNPs were selected because of evidence that the genes they are in are associated with PTSD, depression, substance abuse, HPA-Axis regulation and constituents that influence abnormalities in fear learning or regulation of sleep (see table 1 for SNPs in genes of interest and related citations).
2. **Analysis of Gene Candidates:** Relationships between SNPs and other features including other SNPs was assessed using Fischer’s exact test where each SNP is coded as a 3 level variable (major homozygote, heterozygote, minor homozygote). The procedure is sensitive to determine both the presences of linear relationships (i.e. linear changes in the number of minor alleles) and differences as a three level categorical feature. As such, the presence of a relationship between SNPs and other features can be identified but the nature of the relationship remains obscure in this analytic procedure.^18^ Gene features were constructed by first identifying probabilistic relationships between individual SNPs and both other SNPs and other features and then combining SNPs in a gene to identify all edges between SNPs in a particular gene and features it is connected to. This provides a more interpretable assessment of the relationship between genes and other features but lacks information about the direction of the relationship or the relationship to individual SNPs.

| Gene Name | Gene Symbol | SNPs |
| --- | --- | --- |
| Auxin binding protein 1(70) | ABP1 | rs10273373, rs2052129, rs10156189, rs10156191, rs1049742, rs2301257, rs10893, rs1049748, rs11771771, rs12539, rs2071517, rs4725970 |
| Ankyrin 3(71) | ANK3 | rs9804190 |
| Brain-derived neurotrophic factor(71) | BDNF | rs6265 |
| Catechol-o-methyltransferase(71) | COMT | rs4680 |
| Cholinergic receptor, nicotinic, alpha 5 (71)* | CHRNA5 | rs16969968 |
| Cytoplasmic polyadenylation element-binding protein 1(72) | CPEB1 | rs3850610, rs2871011, rs17158413, rs4779033, rs8032281, rs783539, rs783528, rs12438371, rs783521, rs11635044, rs4778686 |
| Corticotropin releasing hormone receptor 1(73) | CRHR1 | rs16969968, rs110402 |
| C-reactive protein(74) | CRP | rs3091244 |
| CUB and sushi domain-containing protein 1(75) | CSMD1 | rs4875113 |
| Dopamine beta-hydroxylase(71) | DBH | rs1611115 |
| Disrupted in schizophrenia 1(75) | DISC1 | rs6675281 |
| Dopamine receptor D2(71) | DRD2 | rs1799978 |
| Fatty acid amide hydrolase(76) | FAAH | rs324420 |
| FK506 binding protein 5(71) | FKBP5 | rs3800373, rs9296158, rs9470080 |
| Galanin receptor 1(77)* | GALR1 | rs2717162 |
| Glucagon-like peptide 1 receptor(78) | GLP1R | rs2717162, rs1042044 |
| Histidine decarboxylase(58) | HDC | rs17740607, rs860526, rs7182203, rs2238292, rs8029889, rs2853766, rs16963514, rs854151, rs1549521, rs7166052 |
| Histamine N-methyltransferase(58) | HNMT | rs6430764, rs2071048, rs1471003, rs1471001 |
| Histamine Receptor H1(58) | HRH1 | rs346076, rs901865, rs1809049, rs346068, rs346070 |
| Histamine Receptor H2(58) | HRH2 | rs2067474, rs1800689 |
| Histamine Receptor H3(58) | HRH3 | rs3787430, rs3787429 |
| Histamine Receptor H4(58) | HRH4 | rs9944755, rs880263, rs4483927, rs16940765, rs13381219, rs643552, rs11662595, rs9945837, rs1421125, rs475997, rs17797975, rs4800573 |
| lincRNA AC068718.1(71) | lincRNA AC068718.1 | rs10170218 |
| Mineralocorticoid Receptor Gene(73) | MR Gene | rs5522 |
| NF-kappa-B inhibitor alpha(60) | NFKBIA | rs1957106, rs8904, rs2233406, rs3138055, rs1050851 |
| Neuropeptide S(79) | NPS | rs11621961 |
| Neuropeptide Y(71) | NPY | rs16147 |
| Neuronal cell adhesion molecule(75) | NRCAM | rs1269637 |
| Neurotrophic tyrosine kinase receptor type 2(80) | NTRK2 | rs1867283 |
| Neurotensin receptor 1(81) | NTSR1 | rs4334545, rs11117072, rs10863088 |
| Orexin(82) | OXTR | rs13087941, rs2268495, rs2301261 |
| Piccolo presynaptic cytometric protein(83) | PCLO | rs2522833 |
| Rar related orphan receptor A(71) | RORA | rs8042149 |
|  |  | rs406001(84) |
| SCLT1(85) | SCLT1 | rs1433375 |
| Serpin peptidase inhibitor, clade A(71) | SERPINA6 | rs11621961 |
| Solute carrier family 6 member 15(71) | SLC6A15 | rs1545843 |
| Tolloid like 1 (71)* | TLL1 | rs406001 |
| Tyrosine hydroxylase 2(71)* | TPH2 | rs11178997 |
| WW domain-containing protein 1(71) | WWC1 | rs10038727 |
| *Note*: SNPs in the above genes were included in both predictive and causal models with the exception of those marked with an*. These SNPS were found to lack sufficient allele variance in the current population for statistical modeling. | | |

***Table S1*:** Gene Name, gene symbol, and candidate SNPs in genes associated with PTSD, depression, substance use, fear learning, and/or HPA-Axis functioning.

**References**

1. McCaslin, S. E. Trait dissociation predicts posttraumatic stress disorder symptoms in a prospective study of urban police officers. *J. Nerv. Ment. Dis.* **196**, 912-8 (2008).
2. Weissman, M. M. *et al*. Brief screening for family psychiatric history: The family history screen. *Arch. Gen. Psychiatry* **57**, 675-82 (2000).
3. Bernstein-Carlson, E. M. & Putnam, F. W. Development, reliability, and validity of a dissociation scale. *J. Nerv. Ment. Dis.* **174**, 727-35 (1986).
4. Gameroff, M. J., Wickramaratne, P. & Weissman, M. M. Testing the short and screener versions of the social adjustment scale–self‐report (SAS‐SR). *Int. J. Methods Psychiatr. Res.* **21**, 52-65 (2012).
5. Watson, D., Clark, L. A. & Tellegen, A. Development and validation of brief measures of positive and negative affect: The PANAS scales. *J. Pers. Soc. Psychol.* **54**, 1063-70 (1988).
6. Derogatis, L. R. & Unger, R. (2010). Symptom Checklist‐90‐Revised. In the Corsini Encyclopedia of Psychology (eds I. B. Weiner and W. E. Craighead).
7. Bremner, J. D., Bolus, R. & Mayer, E. A. Psychometric properties of the Early Trauma Inventory-Self Report. *J. Nerv. Ment. Dis.* **195**, 211-8 (2007).
8. Shahid, A., Wilkinson, K., Marcu, S. & Shapiro, C. M. Insomnia Severity Index (ISI). *STOP, THAT and One Hundred Other Sleep Scales*: Springer, pp 191-193. 2012
9. Buysse, D. J., Reynolds, C. F., Monk, T. H., Berman, S. R. & Kupfer, D. J. The Pittsburgh Sleep Quality Index: A new instrument for psychiatric practice and research. *Psychiatry Res.* **28**, 193-213 (1989).
10. Spielberger, C. (1988): State-Trait Anger Expression Inventory, Research Edition. Professional Manual. Odessa, FL: Psychological Assessment Resources.
11. Kulka, R. A. *et al*. Assessment of posttraumatic dress disorder in the community: Prospects and pitfalls from recent studies of Vietnam veterans. *Psychological Assessment: A Journal of Consulting and Clinical Psychology* **3**, 547-560 (1991).
12. Weiss, D. S. *et al*. (In submission): The Critical Incident History Questionnaire: A Method for Measuring Total Cumulative Exposure to Critical Incidents.
13. Marmar, C., Metzler, T. & Otte, C. The peritraumatic dissociative experiences questionnaire: In: Wilson JP, Keane TM, editors. *Assessing Psychological Trauma and PTSD*: Guilford Press, 2004.
14. Brunet, A. *et al*. The peritraumatic distress inventory: A proposed measure of PTSD criterion A2. *Am. J. Psychiatry* **158**, 1480-5 (2001).
15. Beck, A.T., Steer, R.A., Brown, G.K., 1996. Beck Depression Inventory—II: Manual. The Psychological Corporation, San Antonio, TX.
16. Conybeare, D., Behar, E., Solomon, A., Newman, M. G. & Borkovec, T. D. The PTSD checklist-civilian version: Reliability, validity, and factor structure in a nonclinical sample. *J. Clin. Psychol.* **68**, 699-713 (2012).
17. Pruessner, J. C. *et al*. Free cortisol levels after awakening: A reliable biological marker for the assessment of adrenocortical activity. *Life Sciences* **61**, 2539-49 (1997).
18. Moore, J. H. & Dunlap, J. C. (2010): *Computational methods for genetics of complex traits* Academic Press.
